# Tailoring of Health-Promotion Video Messaging for Reproductive-Aged Women at Risk for Developing Cardiometabolic Disease: Qualitative Focus-Groups Study

**DOI:** 10.2196/52583

**Published:** 2024-03-05

**Authors:** Jacqueline Kent-Marvick, Bryan Gibson, Alycia A Bristol, Stephanie St Clair, Sara E Simonsen

**Affiliations:** 1 College of Nursing University of Utah Salt Lake City, UT United States; 2 School of Medicine University of Utah Salt Lake City, UT United States

**Keywords:** cardiometabolic disease, type 2 diabetes mellitus, gestational diabetes mellitus, hypertensive disorder of pregnancy, prediabetes, obesity, women’s health, lifestyle change, health promotion technology, qualitative research

## Abstract

**Background:**

Targeting reproductive-aged women at high risk for type 2 diabetes (T2D) provides an opportunity for prevention earlier in the life course. A woman’s experiences during her reproductive years may have a large impact on her future risk of T2D. Her risk is 7 to 10 times higher if she has had gestational diabetes (GDM). Despite these risks, T2D is preventable. Evidence-based programs, such as the National Diabetes Prevention Program (DPP), can reduce the risk of developing T2D by nearly 60%. However, only 0.4% of adults with prediabetes have participated in the DPP to date and reproductive-aged women are 50% less likely to participate than older women. In prior work, our team developed a mobile 360° video to address diabetes risk awareness and promote DPP enrollment among at-risk adults; this video was not designed, however, for reproductive-aged women.

**Objective:**

This study aims to obtain feedback from reproductive-aged women with cardiometabolic disease risk about a 360° video designed to promote enrollment in the DPP, and to gather suggestions about tailoring video messages to reproductive-aged women.

**Methods:**

Focus groups and a qualitative descriptive approach were used. Women with at least 1 previous pregnancy, aged 18 to 40 years, participated in one of three focus groups stratified by the following health risks: (1) a history of GDM or a hypertensive disorder of pregnancy, (2) a diagnosis of prediabetes, or (3) a BMI classified as obese. Focus-group questions addressed several topics; this report shared findings regarding video feedback. The 3 focus-group discussions were conducted via Zoom and were recorded and transcribed for analysis. Deductive codes were used to identify concepts related to the research question and inductive codes were created for novel insights shared by participants. The codes were then organized into categories and themes.

**Results:**

The main themes identified were positive feedback, negative feedback, centering motherhood, and the importance of storytelling. While some participants said the video produced a sense of urgency for health-behavior change, all participants agreed that design changes could improve the video’s motivating effect on health-behavior change in reproductive-aged women. Participants felt a tailored video should recognize the complexities of being a mother and how these dynamics contribute to women’s difficulty engaging in healthy behaviors without stirring feelings of guilt. Women desired a video with a positive, problem-solving perspective, and recommended live links as clickable resources for practical solutions promoting health behavior change. Women suggested using storytelling, both to describe how complications experienced during pregnancy impact long-term health and to motivate health behavior change.

**Conclusions:**

Reproductive-aged women require tailored lifestyle-change messaging that addresses barriers commonly encountered by this population (eg, parenting or work responsibilities). Moreover, messaging should prioritize a positive tone that harnesses storytelling and human connection while offering realistic solutions.

## Introduction

Targeting reproductive-aged women at high risk for type 2 diabetes (T2D) provides an opportunity for prevention earlier in the life course [1]. This is especially important as a woman’s health status during her reproductive years may have a large impact on her future risk of T2D [2-5]. Her risk is 7 to 10 times higher if she has had gestational diabetes (GDM) [2,5]. Despite these risks, T2D is preventable. Evidence-based programs, such as the National Diabetes Prevention Program (DPP), can reduce the risk of developing T2D by nearly 60% [6,7]. However, very few adults with prediabetes participate in the DPP and reproductive-aged women are 50% less likely to participate than older women [8]. Our team earlier developed a 360° video to increase risk perception of T2D. The video helps participants conceptualize T2D complications throughout the lifespan and is designed to promote DPP enrollment among at-risk adults. It was developed with input from Hispanic community members and was not designed specifically for reproductive-aged women [9]. Because few studies have attempted to understand methods motivating reproductive-aged women to engage in healthy lifestyle change, this study aims to obtain feedback from reproductive-aged women with cardiometabolic disease risk factors in order to tailor messaging for this population.

## Methods

### Overview

Designed for middle-aged Hispanic men and women with prediabetes, the 360° video can be viewed with a man’s or woman’s voice and in English or Spanish. Viewers can move their phones around to look at the video’s world [10]. The video depicts an individual who has made unhealthy eating choices and has foregone dental care, thereby increasing their risk for T2D. It shows progression from prediabetes through diabetes leading to a heart attack. The video ends with a statement that diabetes can be prevented through the DPP.

Eligible participants included women who have had at least 1 previous pregnancy, were 18 to 40 years of age, and had one of the following three health risks: (1) history of GDM or hypertensive disorder of pregnancy, (2) diagnosis of prediabetes, or (3) BMI classified as obese. Those with GDM or prediabetes were eligible regardless of BMI. The 3 focus groups were conducted via Zoom by an interviewer from the University of Utah’s Center for Clinical and Translational Science Community Collaboration and Engagement Team (CCET) who had been trained in focus-group best practices and community-engaged research. Focus-group questions enabled key themes relating to the topic to be identified [11]. Focus-group participants were recruited by a CCET study coordinator using flyers. These were posted in the University of Utah cardiologist and obstetrics and gynecology offices, and in Facebook and LinkedIn groups within the CCET’s network. The coordinator screened for eligibility and reviewed the study’s consent cover letter with potential participants. The CCET supports community-engaged research that addresses investigator- and community-identified priorities with the goal of promoting community health needs [12].

Data collection included audio-recording the 1-hour-long focus-group discussions between February and April of 2022. Participants were asked to share their feedback on ways to modify the story, the voice-over content, and the imagery to make the video more impactful and appropriate for reproductive-aged women. The focus-group interviewer prompted participants to provide additional details about their perspectives and experiences. Additionally, the interviewer noted commonalities and differences between participant experiences to foster further insight. Members of the research team were present at the focus groups to answer questions and guide follow-up. The research team had no prior relationship with participants. Audio recordings of the focus groups were transcribed by a research assistant and verified for accuracy by additional team members.

Analysis was completed by 4 team members trained in qualitative methods and women’s health. Qualitative content analysis was used to examine the data. Deductive codes were used to identify concepts related to the research questions (eg, positive and negative feedback). Inductive codes were created for novel insights shared by participants (eg, importance of using storytelling and centering the video’s focus on family relationships). The codes were then organized into categories and themes. Lincoln and Guba’s trustworthiness criteria [13] were used, including weekly coding meetings with authors (AAB, SES, JK-M, and SSC) to discuss nuances in the data to ensure consistency in the application of codes. Coding across transcripts was compared to assess similarities and differences between participant experiences not only as women who had given birth, but also as women with differences in health risks. To address reflexivity during analysis, a group memo document was maintained and reviewed weekly. A member of the team audited group notes and coding to assess for consistency and accuracy. No members had a personal history of hypertensive disorder of pregnancy, GDM, or T2D, but all had clinical and research expertise. Our assumption was that participants would provide critical feedback on the video.

### Ethical Considerations

Before the study’s initiation, waiver-of-consent documentation was received from the University of Utah Institutional Review Board (IRB 00146243). Participants received a consent cover letter containing information about the study’s purpose, and potential risks associated with participation; this information was reviewed with participants before focus-group sessions began, and the voluntary nature of participation was emphasized. Participants received a US $75 gift card for participation in a demographic survey, pre-event Zoom technical session, and focus-group discussion. This amount was suggested by the CCET and was approved and deemed noncoercive by the IRB.

## Results

### Overview

In total, 20 women participated in the 3 focus groups (see Table 1 for demographics).

The main themes were positive video feedback, negative video feedback, centering motherhood, and the importance of storytelling. See Textbox 1 for exemplar quotes.

**Table 1:** Participant demographics (N=20).

Variable		Participants
Age (years), mean (SD)		34.2 (6.46)
Age (years), Range		26-48
**Age (years), n (%)**		
	18-28	6 (30)
	29-39	10 (50)
	40-48	4 (20)
**Race, n (%)**		
	Asian	1 (5)
	Native Hawaiian or Pacific Islander	2 (10)
	White or European	15 (75)
	Multiracial	1 (5)
	Unreported	1 (5)
**Ethnicity, n (%)**		
	Hispanic or Latina	3 (15)
	Non-Hispanic or Latina	17 (85)
**Partnership status, n (%)**		
	Married or living with a partner	15 (75)
	Divorced	2 (10)
	Separated	2 (10)
	Single	1 (5)
**Education, n (%)**		
	Some college	2 (10)
	Associate degree	3 (15)
	Bachelor degree	14 (70)
	Master degree	1 (5)
**Setting, n (%)**		
	Rural	1 (5)
	Suburban	13 (65)
	Urban	6 (30)
**Number of live births, n (%)**		
	1	5 (25)
	2	6 (30)
	3	8 (40)
	4	1 (5)
**Number of children living in home, n (%)**		
	1	5 (25)
	2	8 (40)
	3	6 (30)
	4	1 (5)
**Prior formal lifestyle program attendance, n (%)**		
	Yes	8 (40)
	No	12 (60)
Weight (lbs), mean (SD)		179.025 (40.6740)
Weight, range		125-280

**Themes and exemplar quotes.**

**Positive video feedback**

- *I thought the information was interesting. I knew that, like, eye doctors often catch diabetes, but I wasn’t aware about the dental connection. So, it was information that I... I personally didn’t realize that dentists would give that kind of information about diabetes, or potential risks.*
  [Participant #5; obesity focus group; Hispanic or Latino]
- *When I watched it, I was like, ‘Oh, I need to get my crap together and I don’t want to be that.’ ... I felt somewhat motivated to want to, like, improve my life. Like, I don’t know, but it’s more my personality just to be very direct.*
  [Participant #3, pregnancy complication focus group; White, Asian, non-Hispanic]

**Negative video feedback**

- *I don’t do well with scare tactics. And, so, it’s just... That kind of style is really off-putting to me. It kind of scares me away from it more.*
  [Participant #1; obesity focus group; White, non-Hispanic]
- *I think it lacks empathy. ... While I was watching it, I felt a lot of guilt and shame, um, which is something I think if you’re trying to reach women specifically of childbearing age that that should not be something that I think should be the first thing that ignites in someone.*
  [Participant #7; prediabetes focus group; Hispanic or Latino]

**Centering motherhood**

- *I would much prefer something that was like super fun and encouraging and saying, you know, like, about being excited about having energy to play with my kids, and being around, you know?*
  [Participant #1; obesity focus group; White, non-Hispanic]
- *I don’t think the fear tactic is gonna work. Um, like, that’s like, I feel like that’s somebody that’s like yelling at me to go to the gym, ‘You’re just being lazy.’ Like, ‘Just get up and go.’ Like, ‘Disregard your feelings and your postpartum depression and your postpartum anxiety and all of those other things’.*
  [Participant #7, pregnancy complication focus group; White, non-Hispanic]

**Importance of storytelling**

- *I think our just our innate human beings, like we really love connection. ... What really would have got me, is if I saw like a mother. ... and how she was given that news at the dentist. And saw her progress with her family.*
  [Participant #5; prediabetes focus group; Native Hawaiian or Pacific Islander]
- *Maybe a mother and daughter, where the mother is struggling with the beginnings of these issues and shares the information with her daughter to help prevent from developing issues in the future.*
  [Participant #2, pregnancy complication focus group; White, non-Hispanic]

### Positive and Negative Video Feedback

Feedback about the videos was generally negative. While some participants said the video produced a sense of urgency for healthy behavior change, all participants agreed that it did not motivate health-behavior change in reproductive-aged women.

### Centering Motherhood

The participants felt that a tailored video should convey empathy for the complexities of motherhood and recognize how these dynamics contribute to women’s difficulty engaging in healthy behaviors. Participants emphasized that this tailored messaging should convey urgency. They recommended messages that avoided stirring feelings of guilt. Participants desired a video with a positive, problem-solving perspective. They suggested live links could be embedded within the video as clickable resources for practical solutions, helping to promote health behavior change (eg, links to the DPP website, food-preparation tips, and ways to incorporate children into physical activity).

### Importance of Storytelling

One woman suggested that an optimal approach to the video would summarize a woman’s personal risk from her doctor’s point of view (eg, “you’ve had gestational diabetes, so this is what that means for your long-term health risk”), and then quickly pivot to a problem-solving tone: “Now here’s how we’re going to help you get to where you need to be.” Women across the 3 focus groups agreed that storytelling would be effective in describing how pregnancy complications impact long-term health. For example, a participant suggested the video could include a mother talking with her adult daughter about developing T2D and how she wishes she had acted earlier for prevention. The mother could also encourage her daughter to begin finding solutions while still young and able to maintain health. Other participants agreed that a focus on storytelling and human connection would improve the video’s effectiveness. Finally, the women suggested that it should end by illustrating the benefits of healthy behavior change (eg, being able to keep up with their kids).

## Discussion

In focus group interviews with reproductive women at risk for T2D, participants were enthusiastic about the use of 360° videos to promote healthy-lifestyle change and DPP enrollment, but many found the video to be negative or guilt-inducing. Suggestions for change included positive messages and avoidance of guilt-based messaging. Additionally, participants felt that messaging should acknowledge the time demands and challenges faced by reproductive-aged women, including parenting and work demands, and should convey urgency without creating feelings of guilt.

Few efforts have been made at tailoring diabetes-prevention messaging for reproductive-aged women. An example is the Health-e mums study, which used smartphone app–based messaging to target women with a history of GDM. The app prompted women to improve their health behaviors through goal-setting, and personalized responses to the recording of body weights, diet, and activity. Women provided feedback on messages received through the app. As was true of our study, some Health-e mums participants disliked the use of “negative storytelling,” instead preferring positive, health-coaching messages [14]. Recently, the Centers for Disease Control and Prevention (CDC) released a 1-minute-long promotional DPP video entitled “More Energy Means More Family Fun” [15] that is tailored to reproductive-aged women. It is unclear if the video has been successful in motivating their target population to enroll in the DPP.

Study strengths include participant characteristics (eg, racial or ethnic diversity) and a range of cardiometabolic risk factors. Limitations include the small sample size and the homogeneity of the participants, including the fact that all were mothers. Thus, findings may not apply to women who are not mothers.

In sum, participants highlighted the importance of acknowledging shared experiences and struggles of motherhood, while also offering realistic solutions and a positive tone through storytelling. These findings underscore the importance of tailoring health behavior change messaging to address unique needs of reproductive-aged women and to motivate enrollment in lifestyle change programs. Such messaging is essential for engaging this population in lifestyle change so as to reduce disease burden.

## Abbreviations

CCET: Community Collaboration and Engagement Team
CDC: Centers for Disease Control and Prevention
DPP: Diabetes Prevention Program
GDM: gestational diabetes
IRB: institutional review board
T2D: type 2 diabetes
